# Effects of fatigue on physiological, physical fitness, and stroke performance related parameters in healthy tennis players: a systematic review and meta-analysis

**DOI:** 10.3389/fspor.2025.1578914

**Published:** 2025-04-29

**Authors:** Johanna Lambrich, Thomas Muehlbauer

**Affiliations:** Division of Movement and Training Sciences/Biomechanics of Sport, University of Duisburg-Essen, Essen, Germany

**Keywords:** racket sport, exhaustion, physiology, physical fitness, stroke performance

## Abstract

Fatigue is a multifactorial phenomenon involving central and peripheral mechanisms that could negatively affect performance-related measures in tennis players. The aim of this systematic review and meta-analysis was to quantify the effects of fatigue on physiological (e.g., blood lactate, heart rate), physical fitness (e.g., explosive muscle strength, speed), and stroke performance (e.g., stroke velocity or accuracy) related parameters in healthy tennis players and to provide insight into training and recovery strategies. A systematic literature search of PubMed, Web of Science, and SportDiscus identified studies that examined effects of fatigue in tennis. Inclusion criteria required that studies were conducted with healthy participants, applied fatigue-inducing interventions, and used pre-/post-test designs measuring physiological, physical, or stroke performance related parameters. Effect sizes were calculated using weighted standardized mean differences (*SMD*) to assess the impact of fatigue. The search identified a total of *N* = 642 records. Eighteen trials (318 tennis players) were included. Fatigue evoked large to moderate negative effect on physiological (*SMDw* = −4.19), physical fitness (*SMDw* = −0.74), and stroke performance (*SMDw* = −0.60) related parameters. The larger negative effects of fatigue on physiological and physical parameters compared to stroke performance-related outcomes indicate the importance of targeted recovery strategies (e.g., hydration, nutrition or cold baths). For stroke performance, non-fatigued states are recommended for learning new skills, while practice under fatigued conditions may help to maintain biomechanical efficiency during prolonged games.

## Introduction

Fatigue is generally defined as the loss of the ability to maintain a certain level of performance or strength over an extended period (1). It is a multifactorial phenomenon involving both central and peripheral mechanisms. Central fatigue affects neural control in the central nervous system, reducing signal transmission to the muscles. Peripheral fatigue, on the other hand, refers to muscular processes such as the accumulation of metabolic waste products or reduced calcium release that impair muscle contractility (2, 3). These peripheral impairments directly contribute to motor performance fatigue by reducing the muscle's ability to generate or sustain force during repeated or prolonged efforts. Motor performance fatigue specifically refers to a decline in the neuromuscular system's ability to sustain force production, resulting from both central and peripheral mechanisms. This type of fatigue is critical for understanding the relationship between physiological processes and performance during motor tasks, as it directly affects task performance and endurance (4, 5). Recent evidence (6, 7) suggests that central and peripheral mechanisms do not operate in isolation but interact dynamically during prolonged or high-intensity exercise. For example, peripheral metabolic disturbances may enhance central fatigue through afferent feedback pathways, while central drive may modulate the extent of peripheral muscle activation and fatigue development.

Studies have shown that in tennis players fatigue leads to biomechanical deviations in stroke technique (e.g., ball impact height during serve) and results in a decline in stroke speed and accuracy (8, 9). It has also been shown that fatigue reduces footwork and trunk stability, which in turn impairs the efficiency of the stroke technique (10). At the same time, changes in muscle activation and kinematics can lead to an increased susceptibility to injury, especially during repetitive, high-speed movements such as serves (11, 12). Fatigue has also been shown to increase the error rate of serves and defensive shots (13–15). For example, it has been shown that stroke accuracy decreases by up to 49.6% under high-intensity training, even in experienced tennis players (16). These biomechanical and technical impairments may have a direct impact on match results, as reduced stroke speed and accuracy can increase unforced error rates, limit tactical options, and compromise the ability to execute aggressive plays while fatigued (17). In addition, fatigue can lead to wrong tactical decisions (18).

Although there have been several reviews on fatigue in tennis (18–20), no study has simultaneously examined parameters related to physiology, physical fitness, and stroke performance. Existing reviews tend to focus on isolated aspects—such as the physiological mechanisms underlying fatigue or their biomechanical effects on stroke execution—without examining how these components interact under real-world performance conditions. In competitive tennis, however, fatigue is a multifactorial phenomenon that affects multiple dimensions of performance simultaneously. For example, a reduction in physiological efficiency (e.g., elevated lactate levels or reduced cardiovascular output) can compromise neuromuscular function, leading to impaired movement quality and reduced stroke precision. Therefore, a holistic synthesis is essential to understand how fatigue initiates systemic cascades that contribute to performance decline. While previous reviews have relied primarily on narrative summaries or focused on single domains, the present meta-analysis is the first to quantitatively integrate physiological, physical fitness and stroke performance parameters within a unified analytical framework. In addition, by including subgroup comparisons between elite and sub-elite players, this study provides novel insights into how fatigue responses may vary according to competitive level.

The aim of this systematic review and meta-analysis was to analyze and quantify the effects of motor performance fatigue (4) on physiological parameters, physical performance and stroke performance in healthy tennis players. We hypothesized that performance in all three categories—physiological, physical fitness and stroke performance—would be reduced due to motor fatigue. Furthermore, we expected elite players to show greater resilience to fatigue, particularly in physiological parameters such as blood lactate and heart rate, and physical fitness outcomes such as countermovement jump and sprint performance. The findings of this review may assist coaches and sport scientists in developing more targeted training and recovery strategies that address the specific fatigue-related limitations in physiological, physical fitness and stroke performance parameters. By identifying which areas of performance are most affected by fatigue and how these effects differ between performance levels, practitioners can implement individualized interventions to optimize race readiness, maintain technical execution under pressure and reduce the risk of overuse injuries.

## Methods

### Search strategy

A systematic literature search of the PubMed, Web of Science, and SportDiscus databases was conducted to identify eligible articles. The following Boolean expression was used:

tennis AND (fatigue AND (functional OR performance OR agility OR flexibility OR athletic OR strength OR power OR speed OR fitness OR physical OR reaction time OR stroke OR serve OR forehand OR backhand OR balance OR resistance OR physiology OR heart rate OR blood lactate OR creatine OR cardiocascular)) NOT table

**Figure 1:**
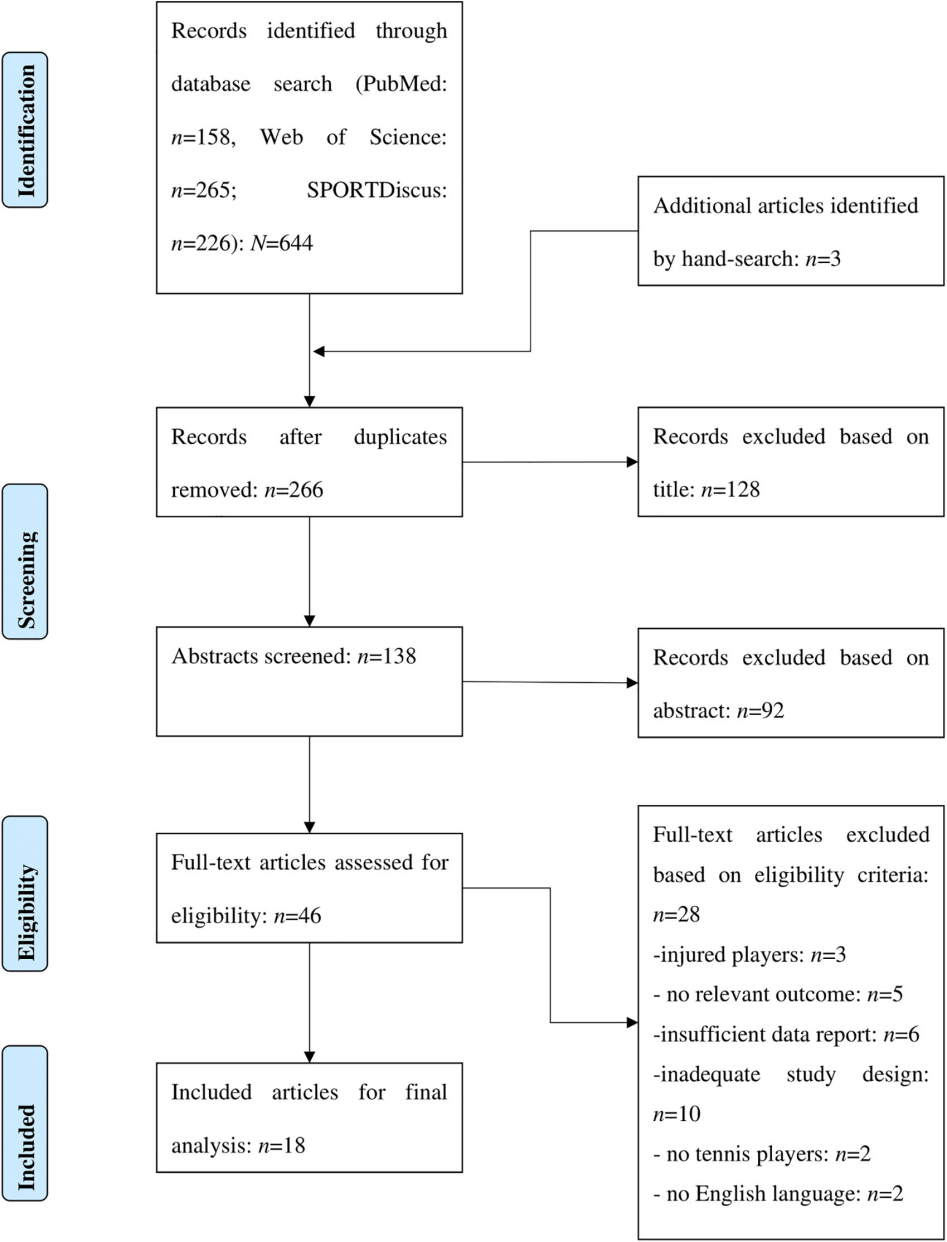
PRISMA flowchart identifying the different phases of the literature search, study selection, and reasons for excluding records.

The search was conducted across the entire history of each database, up to and including January 2025. Only articles written in English with full-text access were included. No search filters regarding publication type, study design, or date were applied in order to maximize the sensitivity and comprehensiveness of the literature search. Furthermore, the reference lists of the included studies and relevant reviews were examined to identify additional eligible articles. After removing duplicates, the titles and abstracts of all retrieved records were evaluated for eligibility based on the inclusion and exclusion criteria independently by both authors (Table 1). The full texts of potentially eligible studies were then assessed independently, with any discrepancies resolved through discussion and consensus. The process of the literature search, study selection, and exclusion of articles is summarized in a PRISMA flow chart (21) (see Figure 1). Disagreements during the study selection process were resolved through discussion, with unresolved cases adjudicated by a third, independent reviewer affiliated with the host institution.

**Table 1:** Overview of the inclusion and exclusion criteria.

Category	Inclusion criteria	Exclusion criteria
Population	Healthy female and male tennis players	Injured tennis players; no tennis players; participants with existing physical or health limitations
Intervention	Studies inducing fatigue, e.g., through repeated sprints, endurance tests, or tennis game simulations; research assessing physiological, physical, or stroke performance of fatigue.	Studies without a motor performance fatigue-related intervention, e.g., cognitive fatigue
Comparison	Pre-/post-test designs	Studies lacking comparative conditions
Outcome	At least one physical fitness, physiological or stroke performance related parameter	Data did not allow calculating effect size
Study design	Experimental studies, cross-sectional studies, or cohort studies, intervention studies with posttest after <24 h	Theoretical work, opinion articles, or studies without primary data or post-testing after more than 24 h

### Study selection criteria

The inclusion and exclusion criteria are summarized in Table 1. Studies were eligible for this review if they (a) included healthy female or male tennis players, (b) implemented a motor performance fatigue-inducing intervention such as repeated sprints, endurance tests, or simulated tennis matches, (c) employed a pre-/post-test design, (d) reported at least one parameter of physiological response, physical fitness, or stroke performance, and (e) conducted interventions with a short-term focus (post-test after less than 24 h). Studies were excluded if (a) injured tennis players, non-tennis players, or participants with physical or health limitations were examined, (b) the intervention lacked a defined method for inducing fatigue, focused solely on long-term adaptations, or addressed only mental fatigue, (c) no pre-/post-test design or comparative condition was present, (d) the data did not allow the calculation of effect sizes, or (e) post-test measurements were conducted after more than 24 h. To ensure that the observed effects reflect acute motor fatigue, only studies with a post-test interval of less than 24 h were included. This decision is supported by the findings of Girard et al. (22), who demonstrated that impairments in physical performance induced by match play were no longer detectable after 24 h of recovery.

### Assessment of methodological quality

The quality of the included studies was assessed using the Joanna Briggs Institute (JBI) Critical Appraisal Tool for Case Series Studies, as described by Munn et al. (23). The tool consists of ten items, each designed to assess critical aspects of methodological quality. These items are answered with “yes”, “no” or “unclear”. Four questions (1, 2, 3, 4) assess inclusion criteria and clarity of case series reporting, focusing on whether inclusion criteria were clearly defined and appropriately applied. Another three questions (5, 6, 7) address the potential for bias in patient selection, whether consecutive cases were included, and the completeness of the clinical information provided. The remaining three questions (8, 9, 10) address the appropriateness of the statistical methods and the reporting of results. Discrepancies in the quality assessment were resolved by a third party, a graduate sports scientist associated with the host institution.

Additionally, Egger's test was applied to assess the presence of publication bias, as it provides a regression-based approach to detect asymmetry in funnel plots. Egger's test is widely used to detect small study effects and publication bias in meta-analyses, although its reliability decreases in cases of high heterogeneity (24). To assess and adjust for potential publication bias, Duval and Tweedie's trim-and-fill method (25) was applied using the R package meta (version 2024.12.0). This method estimates the number of potentially missing studies due to asymmetry in the funnel plot and imputes them to provide an adjusted effect size. In accordance with methodological guidelines, this procedure was only performed for outcomes for which Egger's test indicated significant asymmetry. As the small number of studies within subgroups limits the reliability and interpretability of this approach, the trim-and-fill method was only applied to the overall data sets for each outcome category (26, 27).

### Statistical analyses

All statistical analyses were performed using JASP version 0.19.3.0. To further investigate the effects of fatigue, subgroups were created based on performance level, distinguishing between elite and sub-elite players. In order to quantify the effects of fatigue on physiological parameters, physical fitness and stroke performance in healthy tennis players, the within-subject standardized mean difference (*SMD_W_*) was calculated with the following formula: *SMD_W_* = (pretest mean value − —posttest mean value)/pretest standard deviation (28). The *SMD_W_* can be either positive or negative. A positive value indicates an increase in parameters, expressed by an increase in stroke performance, physical fitness or physiological parameters, from the pretest (non-fatigued) to the posttest (fatigued). Conversely, a negative value indicates a decrease in performance, expressed by a reduction in stroke performance, physical fitness or physiological parameters. *SMD*_W_ values can be classified and interpreted according to Cohen (29) into the following ranges: 0 ≤ 0.49 representing small effects, 0.50 ≤ 0.79 representing moderate effects, and ≥0.80 representing large effects. Further, Deeks et al. (30) postulate that heterogeneity (*I*^2^), which reflects the proportion of variability in study results due to heterogeneity rather than random error, can be interpreted as trivial (0 ≤ 40%), moderate (30 ≤ 60%), substantial (50 ≤ 90%), or considerable (75 ≤ 100%). In cases of substantial or substantial heterogeneity (*I*^2^ ≥ 50%), potential sources were explored using subgroup analyses, leave-one-out sensitivity analysis, and meta-regression models to assess the influence of performance level and study characteristics.

For the meta-analytic approach, the Sidik-Jonkman method was used due to its improved error rates in small sample scenarios and its robustness in estimating heterogeneity (31). Further, the Leave-One-Out (LOO) analysis was conducted to assess the robustness of the results and to identify potentially influential cases (32–34). This method allows for the detection of studies that disproportionately influence the overall effect estimate, increasing the reliability of meta-analytic results. To further investigate the influence of performance level on the overall effect size, a meta-regression was performed including the subgroup as a predictor. Due to methodological differences between subgroup analyses and meta-regression models, slight variations in *SMD* estimates were observed. Specifically, while subgroup analyses estimate effect sizes independently for each group, meta-regression considers subgroup as a covariate in a unified model, which may lead to different weighting of individual studies and slight shifts in overall effect sizes. These differences were expected and are in line with previous methodological recommendations (35). The results of both approaches are reported for transparency. To assess the robustness of the results, a sensitivity analysis was performed by excluding studies identified as influential by the leave-one-out diagnostic. The meta-analysis was repeated without these studies, and changes in effect size and heterogeneity were reported accordingly. In addition to the subgroup (performance level), we conducted exploratory meta-regressions to examine whether the age (youth vs. adults) of the participants or the type of fatigue protocol (e.g., match play, performance tests or conditioning drills) predicted the effect sizes. These additional covariates were tested in separate models to explore their potential influence on outcome variability.

## Results

### Study selection

Figure 1 illustrates the stages of the systematic literature search and study selection process. The initial search identified 644 articles for review and another three studies were included from other sources (e.g., reference lists, review articles). After removing duplicates and screening titles and abstracts, 46 studies were assessed for eligibility. Of these, 28 studies were excluded for the following reasons: three involved injured tennis players, five did not report relevant parameters (e.g., physiology, physical fitness and stroke performance), six lacked sufficient information on outcome measures, ten used inadequate study designs, two did not include tennis players, and two was not written in English.

### Study coding

The included studies were coded in accordance with the following variables to ensure a consistent approach to data extraction: author and year of publication, number of subjects, sex, age, and the study group categorized by the type of fatigue protocol applied. To evaluate the results, three main categories of parameters were differentiated: physical fitness (e.g., counter movement jump, shuttle run), stroke performance (e.g., stroke velocity, stroke accuracy), and physiological response (e.g., blood lactate, heart rate). As some studies reported more than one variable within the same outcome category, we gave priority to the most frequently reported measure in each category to minimize the heterogeneity between studies (Table 2). For physiological measures, blood lactate was selected as the primary outcome, with creatine kinase and vital capacity as alternative measures. For physical fitness, the countermovement jump (CMJ) was the preferred outcome, while knee extension strength, center of pressure (COP) displacement, 20-m shuttle run and *T*-test were considered as alternative measures. For stroke performance, serve velocity was the most reported outcome, with serve speed and serve accuracy used as alternatives when serve velocity was not available. The number of studies using each measure is displayed in Table 2.

**Table 2:** Overview of the preferred and alternative outcome by category.

Category	Preferred outcome	Alternative outcome
Physiology	Blood lactate (*n* = 3)	Creatine kinase (*n* = 1) Vital capacity (*n* = 1)
Physical fitness	Countermovement jump (CMJ) (*n* = 5)	Knee extension strength (*n* = 1) Center of pressure displacement (*n* = 1) 20-m shuttle run (*n* = 1) *T*-test (*n* = 1)
Stroke performance	Serve velocity (*n* = 8)	Stroke velocity (*n* = 2) Stroke accuracy (*n* = 2)

### Study characteristics

This meta-analysis includes 18 studies that investigate the impact of fatigue-inducing interventions, match play, and training on physiological parameters, physical fitness and stroke performance (see Table 3). The studies included a total of 318 tennis players, with sample sizes ranging from 6 (36) to 36 (37) participants. The age of the participants ranged from twelve to 37 years. Significant variation was observed in performance levels across the studies, with participants ranging from professional players (38) to national-level players (8, 36, 39–42), elite (37) and competitive (43) junior players, advanced-level players (9, 12, 44, 45), county players (14) and recreational players (46, 47). Furthermore, one study incorporated a combination of participants with varying degrees of expertise, including both experts and non-experts (16). Additionally, several studies involved mixed-sex cohorts (15, 16, 44, 47, 48) or did not report gender (12, 37), while others (8, 9, 36, 38–43, 45, 46) exclusively recruited male participants.

**Table 3:** Studies examining the effects of fatigue on physiological, physical fitness, and stroke performance related parameters in healthy tennis players.

Reference	No. of participants; sex; age [years (mean ± SD or range)]; performance level	Fatigue protocol	Outcome and unit
Vergauwen et al. (8)	20; M; 21 ± 1 years; national	Leuven Tennis Performance Test	Serve velocity [km/h]
			Shuttle run [s]
Ferrauti et al. (39)	10; M; 25.3 ± 3.7 years; national	Passing shot drill with 10 s rest	Stroke velocity [km/h]
			Blood lactate [mmol/L]
		Passing shot drill with 15 s rest	Stroke velocity [km/h]
			Blood lactate [mmol/L]
Davey et al. (15)	18; M (9), F (9); 19–23 years; county	Loughborough Tennis Skills Test: Groundstrokes	Forehand accuracy [%]
Maraga et al. (36)	6; M; 12.8 ± 1.2; nationally ranked	90 min single match	CMJ [cm]
			Stroke velocity [km/h]
			Creatine kinase [U/L]
Malliou et al. (37)	36; N/A; 14 ± 2 years; elite	90-min training session	Right knee extensors at 60°/s [Nm]
Lyons et al. (16)	13; M (7), F (6); 19.5 ± 3.0 years, experts	Modified Loughborough Tennis Skills Test: Groundstrokes	Groundstroke accuracy [%]
	11; M (13), F (4); 24.9 ± 9.6 years, non-experts	Modified Loughborough Tennis Skills Test: Groundstrokes	Groundstroke accuracy [%]
Murphy et al. (46)	8; M; 24.2 ± 1.2 years; recreational	Cardio tennis session	Blood lactate [mmol/L]
	8; M; 37.7 ± 6.7 years; recreational		Blood lactate [mmol/L]
	8; M; 24.3 ± 2.6 years; recreational		Blood lactate [mmol/L]
	8; M; 35.6 ± 2.7 years; recreational		Blood lactate [mmol/L]
Rota et al. (12)	10; N/A; 23.8 ± 4.0 years; advanced	40-min fatiguing intermittent exercise (4 sets of intense tennis strokes)	Serve velocity [m/s]
Gescheid et al. (40)	7; M; 21.4 ± 2.2 years; national ranking	4-h singles tennis match	Serve velocity [km/h]
			CMJ [cm]
Pialoux et al. (43)	11; M; 13.4 ± 1.3 years; competitive	Playing HIIT session	Blood lactate [mmol/L]
			Serve velocity [km/h]
		Non-playing HIIT session	Blood lactate [mmol/L]
			Serve velocity [km/h]
Gomes et al. (41)	10; M; 16.6 ± 1.4 years; national	3-h match play	CMJ [cm]
Martin et al. (9)	8; M; 20.4 ± 2.8 years; advanced	3-h match play	Serve velocity [m/s]
Moreno-Perez et al. (38)	26; M; 20.4 ± 4.4; professional	Simulated tennis match (best of three)	Serve velocity [km/h]
			Isometric strength IR (dominant side) [N/kg]
Amatori et al. (47)	12; M (8), F (4); 23.0 ± 5.9 years; recreational	120-min match	CMJ [cm]
Colomar et al. (45)	15; M; 16.5 ± 1.5 years; advanced	80-min simulated match	COP displacement [mm]
Fuentes-Garcia et al. (48)	32; F (7), M (25); 21.4 ± 1.5 years; recreational	HIIT training	Serve velocity [km/h]
			CMJ [cm]
			Forced vital capacity [l]
Colomar et al. (44)	20; M (12), F (8); 16.9 ± 1.7 years; advanced	80-min simulated tennis match	Serve velocity [km/h]
			MVC IR [N]
Bilic et al. (42)	21; M; 12.9 ± 0.8 years; national	300-m running test	*T*-test [s]
			Serve precision (1–10)

CMJ, countermovement jump; COP, center of pressure; F, female; HIIT, high intensity interval training; IR, internal rotation, M, male; MVC, maximum voluntary contraction; NA, not available; RM, repetition maximum; *SMDw*, within-subject standardized mean difference.

For subgroup analyses, players were classified as “elite” if they were described as professional, expert, competitive or elite, whereas “sub-elite” included recreational, county, non-expert or advanced players, based on the classifications reported in the original studies.

### Outcome measures

A quantitative synthesis of the literature revealed that a total of five studies analyzed the physiological response to fatigue. Three studies focused on the analysis of blood lactate (42, 43, 46), while one study examined the role of creatine kinase (36) and vital capacity (48), respectively. A total of eleven studies were conducted to investigate the influence of fatigue on physical fitness. Five studies used the CMJ as a measure of physical fitness (36, 40, 41, 47, 48), while two studies assessed internal rotation strength (38, 44). Further, knee extension strength (37), COP displacements (45), a 20-m shuttle run (8), and the *T*-test (42) were each assessed in one study. The impact of fatigue on stroke performance in tennis was evaluated in twelve studies. Of these, eight investigated the effects of fatigue on serve speed (8, 9, 12, 38, 40, 43, 44, 48), two examined its impact on stroke speed (36, 39), and two analyzed its effects on groundstroke accuracy (15, 16).

### Fatigue protocol characteristics

The included studies used different fatigue protocols. Three studies applied a tennis-specific performance test as fatigue protocol. These were the Leuven Tennis Performance Test (8), which measures serve speed and shuttle run performance, or the Loughborough Tennis Skills Test (15, 16) and its modified versions, which assess groundstroke accuracy. In eight trials, simulated match play lasted between 40 min and 4 h (9, 36–38, 40, 41, 44, 45, 47). High intensity interval training (HIIT) tennis sessions were used in one study (43). In addition, passing shot drills with different rest intervals of 10 and 15 s were performed (39). Other protocols included a cardio tennis session (46) and a 40-min intermittent exercise protocol (12). Strength and conditioning interventions included HIIT (48) or a 300-m running test (42).

### Methodological quality of the included trials

#### JBI critical appraisal tool

The quality assessment of the studies included revealed that all studies met ≥3 out of 4 criteria related to the definition and application of inclusion criteria. In addition, all studies met ≥2 of 3 criteria regarding the potential for bias in patient selection, and all studies met ≥2 of 3 criteria addressing the use of statistical methods and reporting of results (Supplementary Table S1). Overall, all included studies met at least seven out of ten criteria.

#### Sensitivity analysis

The LOO sensitivity analysis (Table 4 and Supplementary Table S3) was performed to assess the influence of individual studies on the heterogeneity (*I*^2^) and *SMDw* in physiology, physical fitness, and stroke performance. Exclusion of influential studies resulted in notable changes in effect sizes and heterogeneity values in the different subgroups. In the physiology category, the sub-elite subgroup showed a significant decrease in heterogeneity from considerable (*I*^2^ = 91.97%) to trivial (*I*^2^ = 19.82%) after the exclusion of an influential study (48), while the effect size increased (*SMDw* = −6.85 to −8.46). The elite subgroup remained in the moderate heterogeneity range (*I*^2^ = 31.23%). The overall category retained substantial heterogeneity, with the *I*^2^ decreasing slightly from 97.11% to 95.38% after exclusion. Fuentes-Garcia et al. (48) was identified as an influential study. In the physical fitness category, the elite subgroup showed the most pronounced change, with heterogeneity decreasing from substantial (*I*^2^ = 88.08%) to trivial (*I*^2^ = 19.29%), and the effect size shifting from large (*SMD_W_* = −0.93) to small (*SMD_W_* = −0.01). The sub-elite subgroup showed a reduction in heterogeneity from considerable (*I*^2^ = 91.90%) to substantial (*I*^2^ = 70.62%) and a slight change in effect size (*SMDw* = −0.52 to −0.57). Across all subgroups, Bilic et al. (42) was identified as an important influential study. For stroke performance, the sub-elite subgroup showed a reduction in heterogeneity from considerable (*I*^2^ = 93.92%) to moderate (*I*^2^ = 38.65%) after exclusion, with a corresponding shift in effect size from large (*SMDw* = −0.90) to small (*SMDw* = −0.23). The elite subgroup also showed a reduction in heterogeneity from substantial (*I*^2^ = 56.69%) to moderate (*I*^2^ = 36.69%). Overall, the stroke performance category showed a significant decrease in heterogeneity from substantial (*I*^2^ = 88.61%) to moderate (*I*^2^ = 36.38%). The study by Davey et al. (15) (sub-elite players) and the work by Vergauwen et al. (8) (elite players) were identified as influential.

**Table 4:** Standardized mean difference (SMD) and heterogeneity (*I*^2^) without meta-regression.

Parameter	Group or subgroup	Before exclusion		After exclusion	
		$SMD_{W}$	$I^{2}$	$SMD_{W}$	$I^{2}$
Physiology	Sub-elite players	−6.85	91.965	−8.46	19.824
	Elite players	−1.96	31.228		
	All players	−4.12	97.107	−4.61	95.379
Physical fitness	Sub-elite players	−0.52	91.904	−0.57	70.622
	Elite players	−0.93	88.080	−0.01	19.292
	All players	−0.74	89.860	−0.31	66.549
Stroke performance	Sub-elite players	−0.90	93.923	−0.23	38.646
	Elite players	−0.39	56.691	−0.23	36.696
	All players	−0.60	88.613	−0.23	36.389

#### Meta regression

Meta-regression analysis (Table 5 and Supplementary Table S4) was performed to assess the influence of subgroup classification (elite players *vs.* sub-elite players) on the *SMD* in physiology, physical fitness and stroke performance, both before and after exclusion of influential studies. In the physiology category, subgroup classification had a significant effect before exclusion (*F*_(1,8)_ = 5.460, *p* = 0.048), with the sub-elite group having a significantly lower *SMD* than the elite group (*β* = −4.474, 95% CI = −8.888 to −0.059). After exclusion, this effect became highly significant (*F*_(1,7)_ = 38.155, *p* < 0.001) and the estimated coefficient increased in magnitude (*β* = −6.510, 95% CI = −9.003 to −4.018), indicating a greater difference between the two groups after the removal of influential studies. For physical fitness, no significant effect of subgroup classification was observed before exclusion (*F*_(1,9)_ = 0.324, *p* = 0.583), with the subgroup coefficient (*β* = 0.403, 95% CI = −1.199 to 2.005) showing no meaningful differentiation between elite and sub-elite athletes. After exclusion, the effect remained non-significant (*F*_(1,7)_ = 2.190, *p* = 0.182), with a coefficient of *β* = 0.558, 95% CI = −0.334 to 1.451, indicating no systematic difference between the groups before or after removal of influential studies. In stroke performance, subgroup classification was not a significant predictor of *SMD* before exclusion (*F*_(1,13)_ = 0.606, *p* = 0.450), with a coefficient of *β* = −0.486, 95% CI = −1.834 to 0.862. After exclusion, the effect size became even smaller (*F*_(1,11)_ = 0.00065, *p* = 0.980), with the coefficient approaching zero (*β* = 0.007, 95% CI = −0.618 to 0.632), suggesting no differentiation between groups. In addition to performance level, exploratory meta-regression analyses were conducted to examine the influence of age group (youth vs. adult) and fatigue protocol type (match play, tennis-specific drills, and other protocols) on fatigue-related performance outcomes (Supplementary Table S4). While age did not significantly moderate the effects in any performance category (all *p* > .05), a significant effect of fatigue protocol on physical fitness outcomes was observed after the exclusion of influential studies (*F*_(2,6)_ = 5.718, *p* = .041). *post-hoc* analysis based on estimated marginal means indicated that tennis-specific drills were associated with the strongest fatigue-related reductions in physical fitness [SMD = −1.67, 95% CI (−2.74, −0.60)], compared to match play [SMD = −0.20, 95% CI (−0.60, 0.19)] and other protocols [SMD = 0.09, 95% CI (−0.75, 0.93)].

**Table 5:** Standardized mean difference (*SMD*) and heterogeneity (*I*^2^) with meta-regression.

Parameter	Group or subgroup	Before exclusion		After exclusion	
		$SMD_{W}$	$I^{2}$	$SMD_{W}$	$I^{2}$
Physiology	Sub-elite players	−6.43	N/A	−8.47	N/A
	Elite players	−1.95	N/A		
	All players	−4.19	93.902	−4.85	63.022
Physical fitness	Sub-elite players	−0.52	N/A	−0.58	N/A
	Elite players	−0.93	N/A	−0.02	N/A
	All players	−0.74	90.084	−0.33	61.463
Stroke performance	Sub-elite players	−0.86	N/A	−0.23	N/A
	Elite players	−0.38	N/A	−0.23	N/A
	All players	−0.60	88.519	−0.23	37.675

NA, not available.

#### Egger's test

Egger's test revealed significant asymmetry for physiology (sub-elite players and all players, *p* < 0.001), physical fitness (sub-elite players, *p* = 0.002) and stroke performance (elite players, *p* = 0.002) subgroups. No significant asymmetry was found in the remaining subgroups (Table 6).

**Table 6:** Results for the eggers’ test used to assess publication bias.

Parameter	Group or subgroup	*z*-value	95% CI	*p*-value	Eggers’ test^a^
Physiology	Sub-elite players	−9.636	0.773 to 2.540	<.001	Asymmetry
	Elite players	0.255	−13.218 to 6.709	.799	No asymmetry
	All players	−8.524	0.573 to 2.600	<.001	Asymmetry
Physical fitness	Sub-elite	−3.096	0.528 to 4.046	.002	Asymmetry
	Elite players	0.001	−4.146 to 2.292	.999	No asymmetry
	All players	−1.551	−1.217 to 2.721	.121	No asymmetry
Stroke performance	Sub-elite players	0.236	−5.889 to 3.209	.813	No asymmetry
	Elite players	−3.060	0.190 to 3.162	.002	Asymmetry
	All players	−1.476	−1.323 to 2.830	.140	No asymmetry

95% CI, 95% confidence interval.

^a^ Indicates the presence of publication bias.

#### Trim-and-fill method

Due to the significant asymmetry detected by Egger's test in the physiology category across all players (*p* < .001), a trim-and-fill analysis was performed to adjust for potential publication bias. The unadjusted effect size was SMD = −4.11 [95% CI: (−6.34, −1.88)], with one potentially missing study imputed. After adjustment, the effect size decreased to SMD = −3.54 [95% CI: (−5.93, −1.15)], suggesting a slight overestimation in the original estimate. The corresponding funnel plot is shown in Supplementary Figure S5.

### Effects of fatigue on physiological measures

**Figure 2:**
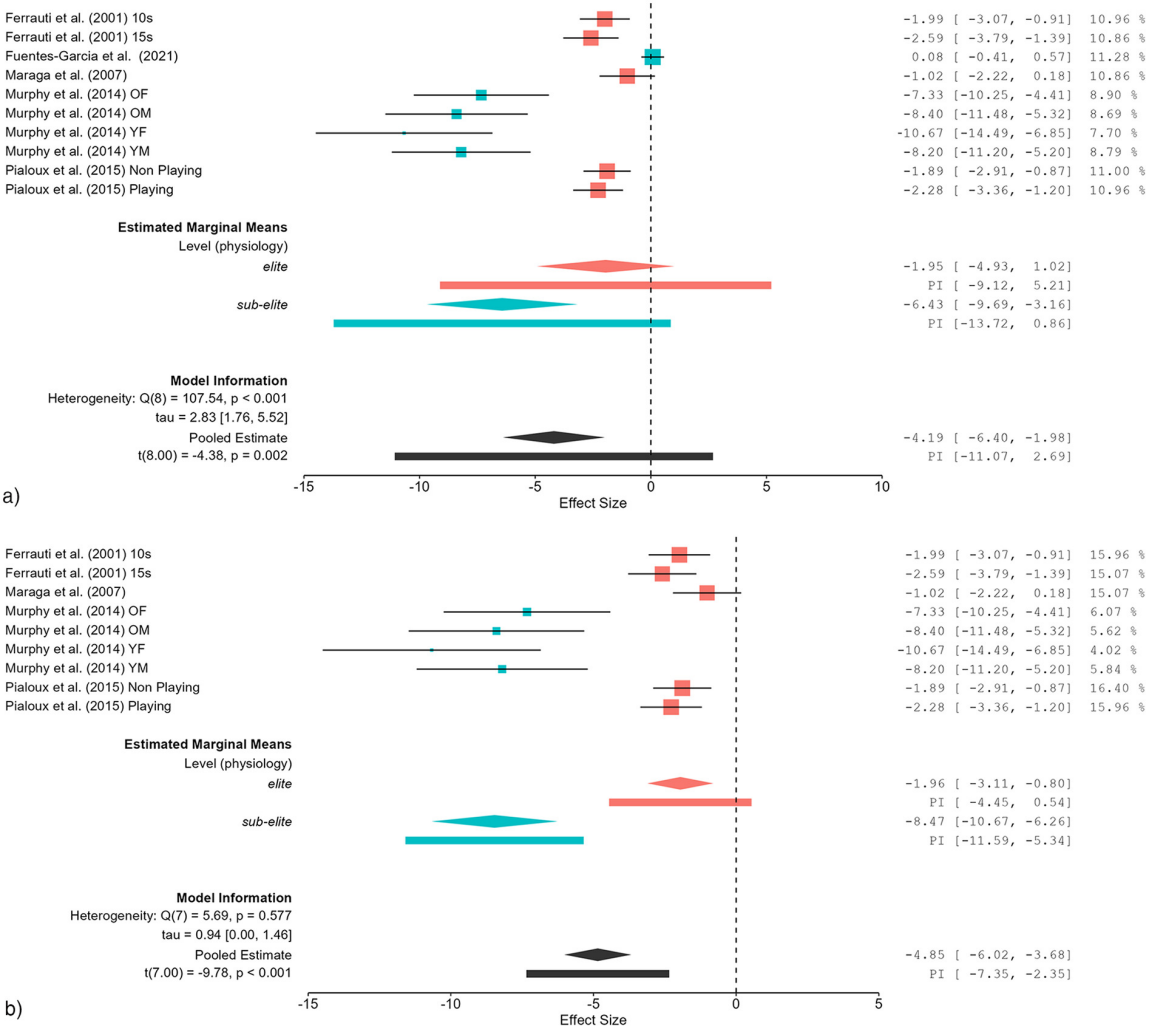
**(a)** Effects of fatigue on measures of physiology (e.g., blood lactate level) in healthy tennis players before study exclusion. OF, older female, OM, older male, YF, younger female, YM, younger male. **(b)** Effects of fatigue on measures of physiology (e.g., blood lactate level) in healthy tennis players after study exclusion. OF, older female, OM, older male, YF, younger female, YM, younger male.

The impact of fatigue on physiological parameters was evaluated through meta-regression analysis (Table 5). As demonstrated in Figure 2a, the sub-elite group showed a large effect (*SMDw* = −6.43) prior to exclusion. After exclusion (Figure 2b), the effect size underwent a further increase (*SMDw* = −8.47), indicating still a large effect of fatigue. For the elite group, the effect size remained stable before and after exclusion (*SMDw* = −1.95 *vs.* −1.96), both indicating large effects. The initial evaluation of the overall physiology category revealed considerable heterogeneity (*I*^2^ = 93.90%), which subsequently diminished to a substantial level (*I*^2^ = 63.02%), accompanied by an augmentation in effect size from *SMDw* = −4.19 to −4.85 following exclusion.

### Effects of fatigue on measures of physical fitness

**Figure 3:**
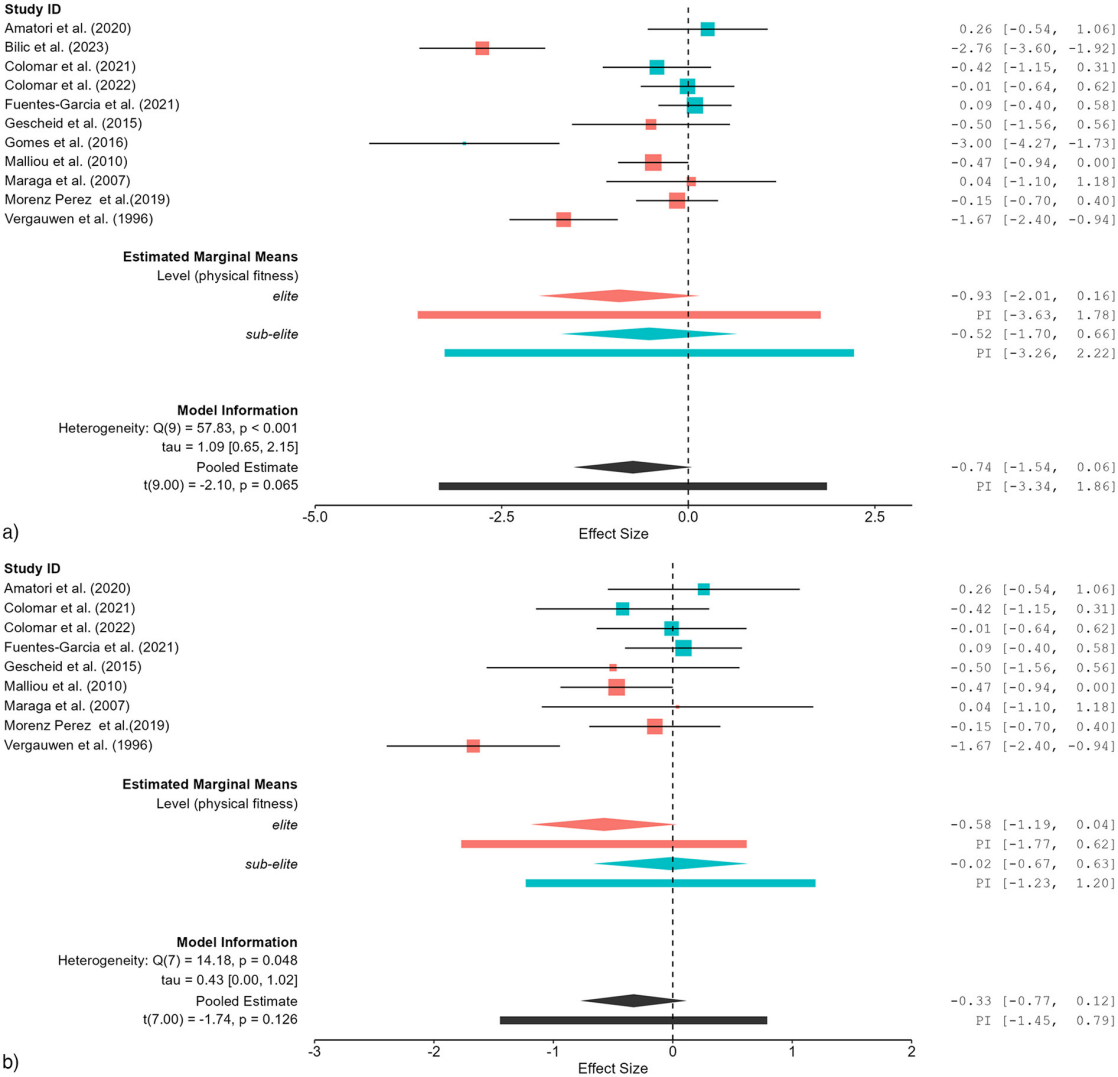
**(a)** Effects of fatigue on measures of physical fitness (e.g., countermovement jump height) in healthy tennis players before study exclusion. **(b)** Effects of fatigue on measures of physical fitness (e.g., countermovement jump height) in healthy tennis players after study exclusion.

The impact of fatigue on physical fitness measures was analyzed through meta-regression (Table 5). Prior to the exclusion of data (Figure 3a), the elite group demonstrated a moderate effect (*SMDw* = −0.93). After the exclusion of data (Figure 3b), the effect size underwent a substantial shift to small (*SMDw* = −0.01). For the sub-elite group, the effect size was moderate before exclusion (*SMDw* = −0.52) and small after exclusion (*SMDw* = −0.33). The overall physical fitness category initially demonstrated considerable heterogeneity (*I*^2^ = 90.08%), which decreased to substantial levels (*I*^2^ = 61.46%), while the effect size shifted from moderate (*SMDw* = −0.74) to small (*SMDw* = −0.31).

### Effects of fatigue on measures of stroke performance

**Figure 4:**
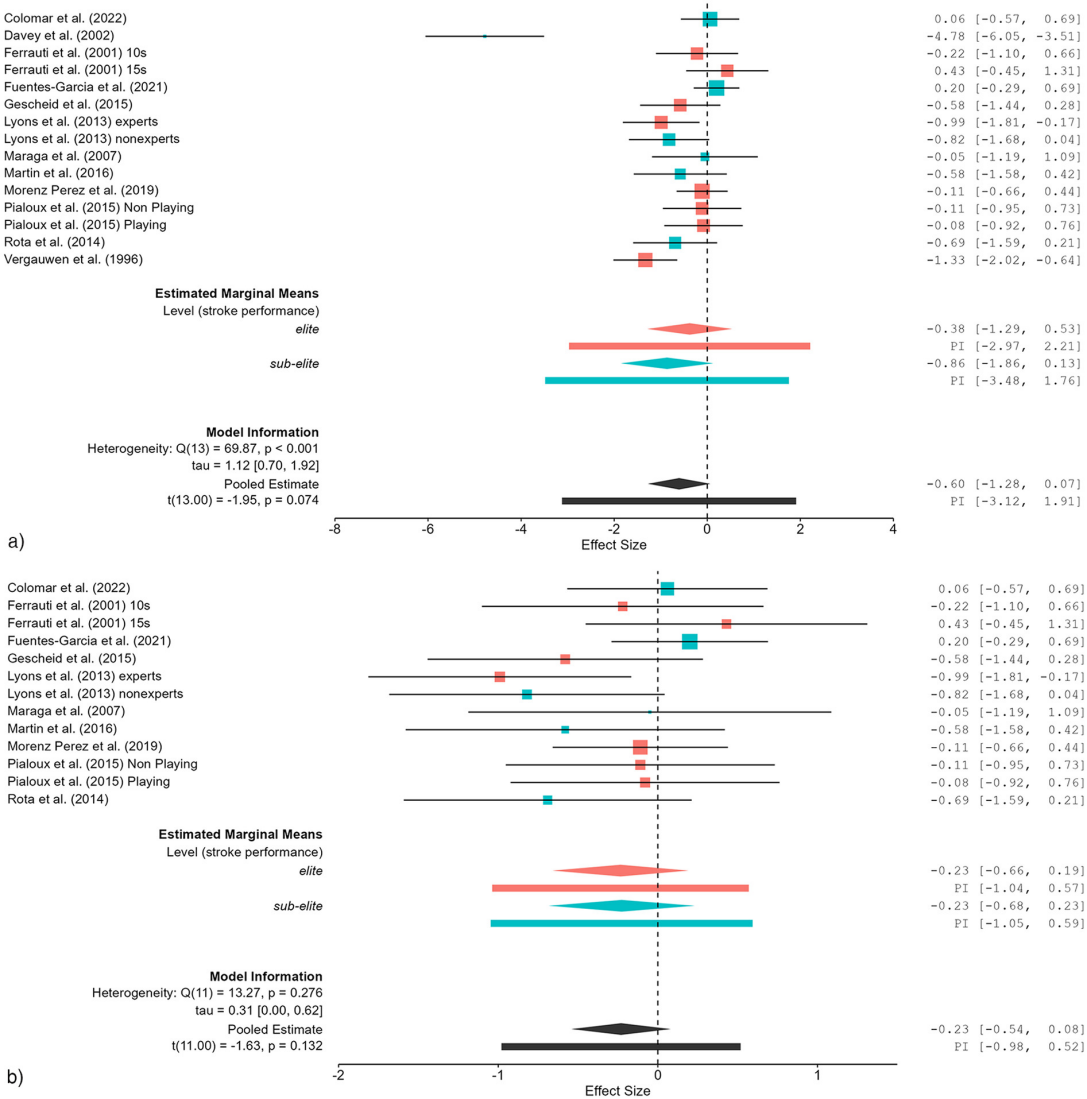
**(a)** Effects of fatigue on measures of stroke performance (e.g., stroke velocity) in healthy tennis players before study exclusion. **(b)** Effects of fatigue on measures of stroke performance (e.g., stroke velocity) in healthy tennis players after study exclusion.

The impact of fatigue on stroke performance was examined using meta-regression (Table 5). Before the exclusion of data, the sub-elite group exhibited a large effect (*SMDw* = −0.86). After data exclusion, the effect size changed to *SMDw* = −0.23, indicating a small effect (Figures 4a,b). For the elite group, the effect size was small before exclusion (*SMDw* = −0.38) and remained small after exclusion (*SMDw* = −0.23). The stroke performance category exhibited substantial heterogeneity (*I*^2^ = 88.52%) at the outset, which diminished to moderate a level (*I*^2^ = 37.67%) following exclusion. The effect size changed from moderate (*SMDw* = −0.60) to small (*SMDw* = −0.23).

## Discussion

The objective of this systematic review and meta-analysis was to investigate the effects of fatigue protocols on physiological, physical fitness, and stroke performance parameters in healthy tennis players. The results demonstrate that fatigue has large negative effects on physiological parameters, moderate effects on physical fitness, and small effects on stroke performance. Furthermore, subgroup analyses revealed that elite players exhibited greater resistance to fatigue compared to sub-elite players, particularly in physiological and physical fitness measures. The exclusion of influential studies resulted in the enhancement of effect sizes in physiology and a substantial reduction in heterogeneity, thereby confirming the robustness of the observed effects.

Fatigue protocols revealed a large effect on physiological parameters with higher blood lactate levels, heart rate, and creatine concentrations. These effects, as well as metabolic disturbances, are also highlighted in studies describing fatigue as a key mechanism for performance decline in tennis (19, 20). The metabolic and thermal stress that threatens physiological stability is particularly elevated during long matches (10). Gescheit et al. (40) showed increasing levels of creatine kinase during 4-h matches for four consecutive days, indicating an accumulation of physiological damage.

Prior to the exclusion of influential studies, the sub-elite group demonstrated heightened fatigue-induced physiological responses in comparison to the elite group, exhibiting an effect size of *SMDw* = −6.43. After the exclusion, the effect size underwent a further augmentation (*SMDw* = −8.47), thereby indicating that the initial analysis may have underestimated the impact of fatigue in this subgroup. Furthermore, heterogeneity decreased from considerable to substantial, thereby supporting the robustness of the findings. The increase in effect size due to the exclusion of studies suggests that some studies may have underestimated the effects of fatigue due to methodological differences, such as shorter protocol duration or reduced training intensity of the participants. Fuentes-Garcia et al. (48) assessed forced vital capacity whereas Murphy et al. (46) measured blood lactate levels. This methodological discrepancy may have contributed to the exclusion of the Fuentes-Garcia et al. (48) study, as blood lactate is a more commonly used marker for fatigue assessment in physiological analyses. Meta-regression confirmed that subgroup classification (elite players *vs.* sub-elite players) significantly influenced physiological effects before exclusion and became even more pronounced after exclusion. These findings imply that higher-trained athletes may possess superior physiological adaptations that enhance fatigue resistance, as previously hypothesized in other studies (16). While fatigue primarily affected physiological responses, its impact on physical fitness parameters was more moderate, with notable variations between elite and sub-elite tennis players, with performance deteriorations being observed in agility time, countermovement jump height, and sprint time. Initially, the elite group exhibited a large effect size (*SMDw* = −0.93), which declined to small (*SMDw* = −0.01) after exclusion. Following the exclusion of studies, the overall *SMDw* was reduced from −0.74 to −0.31, indicating that the effects were small. This finding suggests that influential studies may have overestimated the fatigue-induced performance impairments in the elite subgroup. A possible explanation for this is that Bilic et al. (42) was the only study in this category and subgroup that did not implement a tennis-specific intervention, contributing to methodological differences. This difference in study design may have influenced the estimated effect size, further justifying its exclusion from the sensitivity analysis. For the sub-elite group, effect sizes remained relatively stable before (*SMDw* = −0.52, moderate effect) and after study exclusion (*SMDw* = −0.57, moderate effect). In the overall physical fitness category, heterogeneity was initially considerable (*I*^2^ = 90.08%) but decreased to substantial levels (*I*^2^ = 66.55%) after exclusion. Meta-regression analysis revealed no significant subgroup differences before or after exclusion. These findings indicate that physical fitness parameters are influenced by fatigue, but elite players may mitigate these effects more efficiently through neuromuscular and cardiovascular adaptations (49).

Compared to physiological and physical fitness measures, stroke performance appeared to be the most resilient to fatigue effects (*SMDw* = −0.60), indicating that stroke performance is the most robust parameter of the three outcomes investigated in relation to fatigue, as it is dependent on motor learning (50). However, Rota et al. (12) reported biomechanical adaptations resulting in a reduction in stroke performance and accuracy. In contrast, professional tennis players are able to maintain serve speed over five sets (51). Prior to the implementation of exclusion, the sub-elite group demonstrated a substantial effect (*SMDw* = −0.86), which diminished to a small effect (*SMDw* = −0.23) after the exclusion process. In a similar manner, the elite group initially exhibited a moderate effect (*SMDw* = −0.38), which remained consistent following the exclusion procedure (*SMDw* = −0.23). The stroke performance category exhibited considerable heterogeneity (*I*^2^ = 88.52%) at the outset, which diminished to moderate levels (*I*^2^ = 37.68%) following the exclusion of outlying values. Overall *SMDw* was reduced due to study exclusion to a small effect (*SMDw* = −0.23). The reduction in heterogeneity after exclusion indicates that some studies introduced variability, potentially due to different fatigue assessment methods or player` competition levels. Meta-regression analysis revealed that subgroup classification did not serve as a significant predictor, both before and after the exclusion process. This finding indicates that the effects of fatigue on stroke performance remain relatively stable across a range of competition levels.

Our results showed that physiological parameters (*SMDw* = −4.19) were most negatively affected by fatigue, followed by physical fitness parameters (*SMDw* = −0.74), and stroke performance related parameters (*SMDw* = −0.60). Therefore, physiological recovery should be targeted by specific measures. This can be done through hydration or nutrition during competition (52, 53). Additionally, research indicates that nutritional strategies, including carbohydrate supplementation, can help reduce performance declines caused by fatigue (54). Post-exercise strategies should then be used to promote lactate clearance and accelerate muscle recovery. Studies have shown that strategies such as cold baths, active recovery, and compression garments can aid in this process (55–57). In order to minimize fatigue-effects on physical fitness, high-intensity interval training may be advisable (58). Plyometric training can also improve neuromuscular efficiency, reducing the physiological cost of rapid changes in direction (59). For skill training, care should be taken to ensure that new skills are learned in a non-fatiguing state (60). Specifically, Davey et al. (15) recommend avoiding lactate concentrations >8 mmol/L, heart rates >180 bpm, and perceived exertion >16 during technique training to stabilize tennis skills. On the other hand, technique training under fatigue conditions can be useful in order to maintain biomechanical efficiency under load and to keep stroke speed and accuracy stable over a longer period of time (12, 61). The various differences in performance during training and recovery should also be taken into account. Elite players, who typically have higher neuromuscular efficiency and autonomic recovery capacity (62), benefit from precision recovery protocols (e.g., whole-body cryotherapy, active recovery, compression garments) to maintain high training frequency and manage accumulated load (63). In contrast, sub-elite athletes often show greater declines in performance with fatigue and may require fundamental improvements in anaerobic and strength capacity through high-intensity interval training (HIIT), plyometrics, and neuromuscular coordination work (64). In addition, individualized periodization models—particularly for sub-elite athletes—should ensure sufficient recovery time and controlled exposure to fatigue to support technical learning without performance collapse (65).

### Limitations

The present meta-analysis has several limitations. First, the number of studies per category is relatively small (*n* = 5–12), which limits the statistical power and robustness of the findings. Future replication studies with larger samples are needed to confirm and generalize the observed effects. Secondly, methodological differences between fatigue protocols (e.g., match play *vs.* local fatigue) make direct comparisons difficult. While local fatigue protocols offer high internal validity by minimizing external variables, they often lack ecological validity. In contrast, match play protocols better reflect real game conditions but introduce uncontrolled factors. An integrated approach, such as replicating match-play situations in controlled laboratory settings (e.g., using virtual reality), may help to bridge this gap. Third, heterogeneity in participant characteristics (e.g., gender, age, and skill level) increases the variability of results. Due to limited data, it was not possible to differentiate by gender or age; instead, subgroup analyses based on performance level (elite *vs.* sub-elite) were more appropriate. Fourth, Egger's test indicated potential publication bias and small study effects, particularly for physiological and physical fitness outcomes. Future studies could mitigate this by pre-registering protocols and increasing sample sizes to ensure more balanced evidence. Furthermore, as all included studies focused on short-term responses (<24 h), evidence on long-term or chronic fatigue adaptations remains unexplored—highlighting the need for longitudinal designs. Finally, the under-representation of female athletes limits the ability to draw gender-specific conclusions. Given the known sex differences in fatigue resistance and recovery patterns, future research should explicitly address female-specific responses in tennis-related fatigue.

## Conclusions

This meta-analysis confirms that fatigue exerts the most significant effects on physiological parameters, followed by physical fitness and stroke performance. The exclusion of influential studies resulted in effect sizes that were more pronounced and reduced heterogeneity, suggesting that some studies may have overestimated the fatigue effects, particularly in the sub-elite group. In contrast, elite players demonstrated greater resistance to fatigue, especially in physiological and physical fitness measures, thereby supporting the notion that training adaptations play a crucial role in fatigue management. Future research should explore longitudinal training interventions to enhance fatigue resistance, particularly in sub-elite tennis players, and investigate optimal recovery protocols for physiological stabilization in competitive settings.

## Data Availability

The original contributions presented in the study are included in the article/Supplementary Material, further inquiries can be directed to the corresponding author.
