# Supplementary figures and images for: Effects of fatigue on physiological, physical fitness, and stroke performance related parameters in healthy tennis players: a systematic review and meta-analysis
Source: Front Sports Act Living. 2025 Apr 29;7:1578914. doi: 10.3389/fspor.2025.1578914 (PMC12069318; doi:10.3389/fspor.2025.1578914)

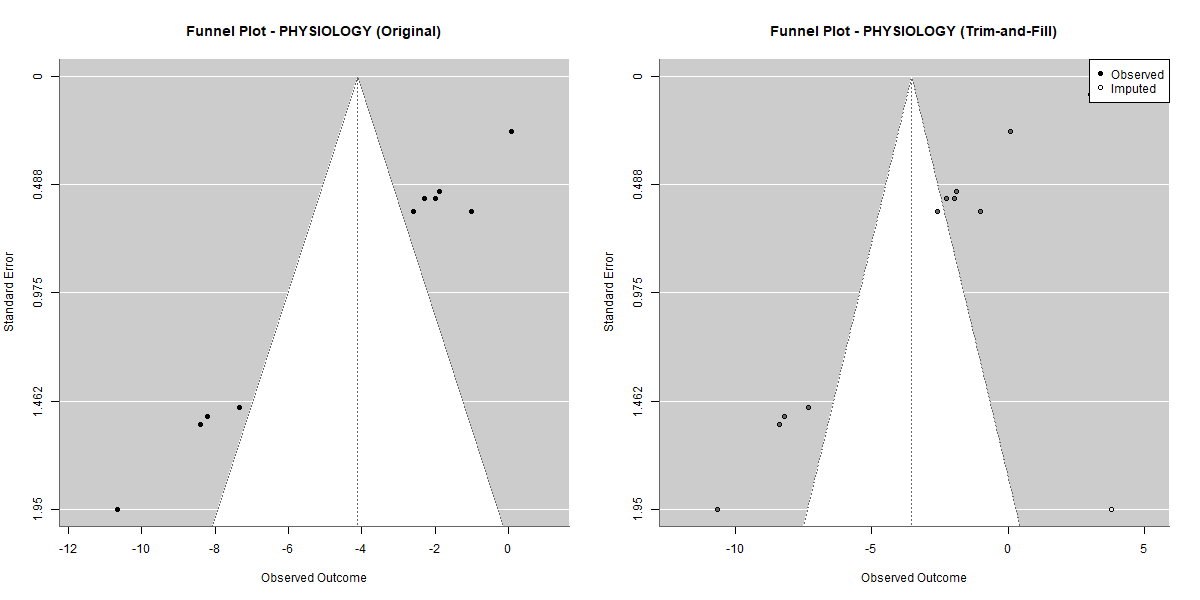

Supplement: Supplementary file 5 [file Image1.png]
